# The effects of dietary energy level on the growth performance of yaks (*Bos grunniens*) were studied based on omics technique

**DOI:** 10.3389/fmicb.2025.1621581

**Published:** 2025-08-12

**Authors:** Yahui Jiang, Jiali Zhang, Kaiwen Wang, Hengbo Feng, Yuting You, Peng Dai, Zhisheng Wang, Rui Hu, Quanhui Peng, Huawei Zou, Jianxin Xiao, Lizhi Wang, Bai Xue

**Affiliations:** ^1^College of Animal Science and Technology, Sichuan Agricultural University, Chengdu, China; ^2^Key Laboratory of the University in Cattle Low Carbon Breeding and Safety Production in Sichuan Province, Animal Nutrition Institute, Sichuan Agricultural University, Chengdu, China

**Keywords:** yak (*Bos grunniens*), dietary energy level, serum metabolome, growth performance, microbiota

## Abstract

**Introduction:**

The yak (Bos grunniens) is uniquely adapted to the Qinghai–Tibet Plateau, yet nutritional guidelines for yaks remain limited and often follow cattle standards. This study aimed to clarify dietary energy requirements to improve yak feeding strategies.

**Methods:**

Fattening yaks were assigned to three diets with different net energy for gain (NEg): low (LE, 4.06 MJ/kg), medium (ME, 4.46 MJ/kg), and high (HE, 4.87 MJ/kg), with 13% crude protein constant. Growth performance, apparent digestibility, serum biochemistry, hormones, and rumen fermentation were measured. Additional, 16S rDNA sequencing analyzed the rumen and fecal microbiota, and serum non-targeted metabolomics profiling was performed.

**Results:**

Results showed that ME and HE diets significantly increased average daily gain and nutrient digestibility while reducing feed conversion ratios (*P* < 0.05). Serum glucose, triglycerides, and growth-related hormones were higher in ME and HE groups, while non-esterified fatty acids was significantly decreased (*P* < 0.05). Higher energy diets increased microbial protein and reduced ruminal ammonia nitrogen. The ME diet increased ruminal *Proteobacteria*, enhancing fermentation and soluble carbohydrate utilization, while promoting fiber-degrading *UCG-005*. High energy diets elevated fecal *Spirochaetota* and *Treponema* abundances. Metabolomics revealed differences mainly in lipidrelated metabolites correlated with specific microbial taxa.

**Discussion:**

In conclusion, increasing dietary energy improves growth, nutrient utilization, and beneficial microbiota profiles in yaks. A medium-energy diet (NEg: 4.46 MJ/kg) optimizes fermentation and microbial balance, providing a scientific basis for precise nutritional strategies in yak production on the Qinghai–Tibet Plateau.

## 1 Introduction

The yak (*Bos grunniens*) is a rare species of cattle primarily distributed across the Qinghai-Tibet Plateau and adjacent alpine and subalpine regions. This species plays a vital role in the livelihoods and socio-economic development of local herding communities, earning designations such as “the boat of the plateau” and “all-purpose livestock” due to its multifaceted utility (Ma et al., 2013; Xue et al., 2018). Globally, the yak population is estimated to be approximately 16 million, with China accounting for over 15 million individuals, representing more than 90% of the global population (Zhang et al., 2020).

The Qinghai-Tibet Plateau, located between 26 and 39°N latitude and 73 and 104°E longitude, has an average elevation exceeding 4,000 m. This region is characterized by low atmospheric oxygen levels, intense solar radiation, abundant sunlight, pronounced diurnal temperature fluctuations, and distinct seasonal differences between cold and warm periods (Zhang et al., 2019). The harsh climatic conditions frequently result in limited forage availability. In pastoral areas, yak breeding predominantly follows a traditional grazing system that is heavily dependent on weather conditions, with grazing practices typically synchronized with the seasonal changes. During the prolonged cold season, yaks often experience extended periods of undernutrition or semi-starvation, which can lead to severe fat depletion and, in extreme cases, mortality (Guo et al., 2021). To address these challenges, alternative feeding strategies-such as combined grazing with supplemental feeding and indoor feeding systems, have been increasingly explored to enhance yak productivity (Cui et al., 2020; Zhu et al., 2022). Researchers have also developed models to investigate the nutritional requirements of yaks (Hao et al., 2020; Zhang, 2022). However, a standardized global reference for yak nutrition remains unavailable, resulting in dietary formulations that are often extrapolated from nutritional standards established for beef cattle, despite notable physiological differences between the two species. Yaks possess a unique ability to tolerate coarse forage and hypoxia conditions, leading to physiological adaptations that distinguish them from beef cattle (Qiu et al., 2015; Liu X. et al., 2023). Therefore, the establishment of nutritional standards specifically tailored to yaks is imperative. To elucidate the energy requirements of yaks raised in pastoral regions, the present study formulated total mixed rations (TMRs) with three distinct NEg levels, based on both previous findings from our laboratory and published dietary energy levels for indoor-fed yaks. The study aimed to investigate the effects of varying energy levels on nutrient digestibility, rumen fermentation parameters, and serum energy metabolites in indoor-fed yaks. Additionally, 16S rDNA sequencing and untargeted metabolomic analyses were employed to examine the effects of dietary energy levels on yak growth performance and rumen fermentation. The study further explored the correlations between the rumen microbial community and differential serum metabolites, as well as the interplay between rumen and fecal microbiota. This research aims to provide foundational data to support the development of evidence-based nutritional strategies for improving the health and productivity of yaks on the Qinghai-Tibet Plateau.

## 2 Materials and methods

### 2.1 Study site and animal ethics

The experiment was conducted at the Larima National Cattle Farm, located in Xinlong County, Ganzi Prefecture, Sichuan Province, China (30.23–31.32°N, 99.37–100.54°E) at an altitude of 3,600 meters. The study period extended from February to April 2024, during which the average daily temperature was approximately −10°C. All animal use protocols and experimental procedures were approved by the Animal Care and Use Committee of the Laboratory Animal Center of Sichuan Agricultural University (Approval No. SCAUAC202402-4).

### 2.2 Experimental design and diet

Twenty-seven healthy male *Maiwa* yaks (48 months of age), with an average body weight of 249.10 ± 31.93 kg, were selected for this study. One week prior to the experiment, cattle pens were treated with insecticidal powder for deworming purposes. The yaks were randomly divided into three treatment groups using a randomized block design, with each treatment consisting of three replicates, and three yaks assigned to each replicate. Each pen had an area of approximately 20 m^2^ (5 m in length and 3.8 m in width). The yaks were fed pelleted total mixed ratios formulated with three different NEg levels: A low energy group (LE: 4.06 MJ/kg), a medium energy group (ME: 4.46 MJ/kg), and a high energy group (HE: 4.87 MJ/kg), while maintaining a constant crude protein (CP) content of 13.0% across all diets. The diets were provided in two equal portions daily, at 09:00 and 17:00. The three diets, ranging from the lowest to the highest energy levels, provided 1.0, 1.1, and 1.2 times the NEg requirements for growing cattle as per the Agricultural Industry Standard of the PRC (2004). The NEg level in the LE group was set based on a reference body weight of 200 kg and an ADG of 0.8 kg. The composition and nutritional content of the experimental diets are presented in Table 1. The experiment employed a one-way ANOVA (Analysis of Variance) experimental design, and included a 7-day adaptation period followed by a 60-day measurement period (days 0–60).

**TABLE 1:** Ingredients and chemical composition of the experimental diets.

Item	Dietary NEg levels, MJ/kg		
(Content,%)	LE: 4.06	ME: 4.46	HE: 4.87
Ingredients (% DM)			
Corn	21.18	31.08	38.70
Cottonseed meal	3.85	5.50	6.30
Soybean meal	5.50	6.32	7.02
Dry liquor lees	9.24	4.18	1.92
Wheat bran	11.28	3.79	1.38
Soybean oil	0.77	0.94	1.20
NaHCO_3_	0.83	0.83	0.90
CaHPO_4_	0.27	0.27	0.30
CaCO_3_	0.44	0.44	0.48
NaCl	1.10	1.10	1.20
1% Premix^1^	0.55	0.55	0.60
Full corn silage	25.00	25.00	20.00
Oat hay	20.00	20.00	20.00
Total	100.00%	100.00%	100.00%
Chemical composition (%)			
NEg^2^, MJ/kg	4.06	4.46	4.87
CP	12.99	12.98	13.01
NDF	40.13	36.01	31.68
ADF	23.01	20.87	18.35
Ca	0.45	0.45	0.45
P	0.43	0.36	0.37
EE	2.99	3.60	4.17

NEg, net energy for gain; LE, low energy; ME, medium energy; HE, high energy. CP, crude protein; DM, dry matter; NDF, neutral detergent fiber; ADF, acid detergent fiber; EE, ether extract; Ca, calcium; P, phosphorus.

^1^The premix was provided following as per kilogram: Cu 14.93 mg, Fe 60 mg, Zn 50.03 mg, Mn 30.02 mg, I 0.80 mg, Se 0.35 mg, Co 0.50 mg, vitamin A 7000 IU, vitamin D_3_ 2000 IU, vitamin E 14 IU, nicotinamide 10 mg, biotin 0.02 mg.

^2^The NEg was calculated according to the tables of feed composition and nutritive values in China (Xiong et al., 2022).

### 2.3 Experimental procedures and sample collection

All yaks were weighed using a weighbridge (Shanghai Yaohua Weighing System Co. in Shanghai, China) prior to feeding on days 0 and 60 of the experiment period. Feed samples (200 g) were collected on days 1, 30, and 60, and stored at –20° for subsequent analysis. Throughout the experimental period (days 1–60), the daily feed intake and feed refusals for each replicate within each treatment group were recorded.

From days 55 to 60, fecal samples (200 g per day) were collected from each group using plastic pans placed beneath the animals. The total fecal output was weighed, and a subsample (100 g) was placed in self-sealing bags and stored at –20°C for later analysis of nutrient digestibility. Additionally, fecal samples (5 g) were collected directly from the rectum of each yak and stored at –80°C in sterile centrifuge tubes for subsequent microbiota analysis.

On day 60, approximately 150 mL of rumen fluid was collected from each yak using an oral stomach tube (Kelibo Equipment Co., Ltd., Wuhan, China) at three time points: 0, 2, and 6 h post-morning feeding. To minimize saliva contamination, the initial 50 mL of rumen fluid was discarded. The pH of the rumen fluid was measured immediately using a pH meter (pH 828-10, Zhongxing Weiye Instrument Co., Ltd., China). Subsequently, the rumen fluid was filtered through four layers of cheesecloth and divided into four aliquots in 50 mL centrifuge tubes, which were subsequently stored at –80°C for further analysis. For the analysis of volatile fatty acids (VFAs), 10 mL of rumen fluid was mixed with 25% metaphosphate acid in a 4:1 volume ratio. For ammonia nitrogen (NH_3_-N) determination, 5 mL of rumen fluid was mixed with methanol at a 1:1 volume ratio. The remaining rumen fluid was stored in two 10 mL centrifuge tubes at –80°C for the analysis of Microbial Protein (MCP) and rumen microbiota.

On day 60, prior to the morning feeding, blood samples (20 mL) were collected from the jugular vein of each yak using evacuated tubes without anticoagulant (Honghu Taining Medical Equipment Co., Ltd., Honghu, China). The samples were kept on ice for 1 h and then centrifuged at 3,000 g for 15 min to separate the serum. The serum samples were stored at –80°C for analyses of serum biochemical parameters, energy metabolism-related hormones, and untargeted metabolomics.

### 2.4 Index determination

#### 2.4.1 Growth performance

Prior to the morning feeding on days 0 and 60 of the experimental period, all yaks were weighed to record their initial body weight (IBW) and final body weight (FBW). The ADG was subsequently calculated based on these measurements. Daily feed intake was recorded throughout the experiment period, and the dry matter intake (DMI) was determined according to the dry matter content of the feed. The FCR was calculated as described by Ahmat et al. (2021).

#### 2.4.2 Apparent digestibility of nutrients

Feed and fecal samples were dried in a forced air oven (DHG-9140A, Changzhou, China) at 65°C for 72 h, followed by equilibration at room temperature overnight. The dried samples were ground to pass through a 1 mm sieve and stored in a self-sealing plastic bag until analysis. Dry matter (DM) content was determined by drying the samples at 105°C for 24 h in a forced air oven. Organic matter (OM) content was assessed by ashing the samples in a muffle furnace at 550°C for 6 h, following AOAC (2006) method 925.45. Total nitrogen content in the feed and feces samples was measured using the micro Kjeldahl method (K1100, Hanon instruments, Jinan, China), and CP content was calculated by multiplying the nitrogen content by 6.25. Neutral detergent fiber (NDF) and acid detergent fiber (ADF) were determined using an automatic fiber analyzer (Ankom Technology, Fairport, NY, United States) according to the methods described by Van Soest et al. (1991) and Robertson and VanSoest (1981). Ether extract (EE) was analyzed by extracting the samples with petroleum ether at 70–85°C for 8 h (Thiex et al., 2003). Phosphorus and calcium content were determined following the standards of the P.R.C. (GB/T6437-2002, GB/T6436-2002, respectively; General Administration of Quality Supervision, Inspection and Quarantine of the People’s Republic of China, 2002a, b). Crude fiber (CF) was measured according to the AOAC method (AOAC, 2020). The apparent digestibility of nutrients was calculated using acid-insoluble ash (AIA) as an internal marker in both feed and fecal samples, following the method described by Block et al. (1981). AIA was determined by treatment with 15% hydrochloric acid according to the Chinese national standard (GB/T 23742-2009, China National Standard, 2009). The formula used for calculating the apparent digestibility of nutrients was as follows:


ApparentDigestibility(%)=100*

[1-(AIAcontentinfeed/AIAcontentinfeces)×

(nutrientcontentinfeces/nutrientcontentinfeed)].

#### 2.4.3 Rumen fermentation parameters

The concentration of VFAs in the rumen fluid was measured using gas chromatography (Abuelfatah et al., 2016), with an Agilent GC-8890 Gas Chromatograph (Agilent Technologies, United States). The concentration of NH_3_-N was determined using the phenol and sodium hypochlorite colorimetric method (Broderick and Kang, 1980). For the determination of MCP, the rumen fluid was first centrifuged, and sodium hydroxide was added to the supernatant, followed by heating in a 100°C water bath (Makkar et al., 1982). The MCP content was then quantified using a protein quantification kit from Nanjing Jiancheng Bioengineering Institute. The levels of rumen NH_3_-N and MCP were analyzed using colorimetric techniques, following the procedures developed by Hristov et al. (2001) and Oosta et al. (1978).

#### 2.4.4 Serum biochemical and energy metabolism indexes

Serum concentrations of glucose (GLU), total cholesterol (TC), albumin (ALB), globulin (GLB), total protein (TP), aspartate transaminase (AST), alkaline phosphatase (ALP), alanine aminotransferase (ALT), urea nitrogen (UN), triglyceride (TG), and lactate dehydrogenase (LDH) were measured using an automatic biochemical analyzer (Hitachi 3100, Hitachi High-Technologies Corporation, Tokyo, Japan). Serum levels of insulin (INS), growth hormone (GH), leptin (LEP), thyroxine (T4), norepinephrine (NE), β-hydroxybutyric acid (β-HB), non-esterified fatty acids (NEFA), glucagon (GC), and insulin-like growth factor-1 (IGF-1) were determined using enzyme-linked immunosorbent assay (ELISA) kits (Lab systems Multiskan MS Type 96, Shanghai Kexing Trading Co., Ltd.).

#### 2.4.5 Determination and analysis of rumen and fecal microbiota

Total microbial genomic DNA was extracted from rumen fluid and fecal samples using the CTAB method. DNA integrity and concentration were assessed by 1.0% agarose gel electrophoresis and a NanoDrop 2000 spectrophotometer (Thermo Scientific, United States). The DNA samples were stored at –80°C until further analysis. The V3–V4 hypervariable region of the 16S rDNA gene was amplified using barcoded primers 341F (5±-CCTAYGGGRBGCASCAG-3±) and 806R (5±-GGACTACNNGGGTATCTAAT-3±). Each PCR reaction (30 μ L total volume) contained 0.2 μ L of each primer (1 μ mol/μ L), 10 μ L of template DNA (1 ng/μ L), 15 μ L Phusion High-fidelity PCR Master Mix (New England Biolabs), and ddH_2_O. The thermal cycling conditions were as follows: 98°C for 1 min; 30 cycles of 98°C for 10 s, 50°C for 30 s, 72°C for 30 s; followed by a final extension at 72°C for 5 min. PCR products were separated by 1% agarose gels electrophoresis, and target fragments were purified using the MinElute Gel Extraction Kit (Qiagen, Germany). The purified amplicons were quantified using the QuantStudio 12K Flex system (Thermo Fisher Scientific, United States), pooled in equimolar concentrations, and sequenced on the Illumina NovaSeq 6000 platform (Illumina, United States).

Raw sequencing data were subjected to quality control using Fastp (v0.23.1), merged with FLASH (v1.2.11), and processed with Cutadapt for adapter removal. Denoising, chimera removal, and amplicon sequence variants (ASVs) inference were conducted using the DADA2 algorithm within the QIIME2 platform. Taxonomic assignment of representative ASVs was performed using the SILVA 138.1 reference database. To ensure comparability across samples, ASVs abundance data were normalized to the sample with the lowest sequencing depth. Taxonomic compositions at the phylum and genus levels were visualized in SVG format using Perl scripts. Venn diagrams of shared and unique ASVs were generated using the VennDiagram package in R. Alpha and beta diversity metrics were calculated and analyzed within the QIIME2 environment.

#### 2.4.6 Determination and analysis of serum metabolites

Serum metabolite profiling was conducted using a liquid chromatography–mass spectrometry (LC-MS) platform (Q Exactive™ HF, Thermo Fisher Scientific, Germany) as described by Want et al. (2006) and Barri and Dragsted (2013). Chromatographic separation was carried out using a Hypersil GOLD column (100 × 2.1 mm, 1.9 μ m; Thermo Fisher Scientific, United States). The mobile phases consisted of solvent A (0.1% formic acid in water) and solvent B (0.1% formic acid in acetonitrile), with the following gradient elution program: 0–1.5 min, 98% A/2% B; 1.5–3 min, 15% A/85% B; 3–10 min, 0% A/100% B; 10–12 min, re-equilibration to 98% A/2% B. The flow rate was maintained at 0.2 mL/min, and the column temperature was set at 40°C. Mass spectrometric detection was performed using electrospray ionization (ESI).

Raw LC-MS data were preprocessed using Compound Discoverer 3.3 (Thermo Fisher Scientific), including peak alignment, peak extraction, and quantification, with filtering based on retention time and mass-to-charge ratio (m/z). Metabolite identification was performed by matching MS/MS spectra against high-resolution databases (mzCloud, mzVault), and the MassList library. Only metabolites with a coefficient of variation (CV) of less than 30% in quality control (QC) samples were retained for subsequent analysis (Dai et al., 2017). Data quality was evaluated by calculating Pearson correlation coefficients between QC samples based on the relative quantification of detected metabolites (Rao et al., 2016). Furthert data processing, including data normalization, principal component analysis (PCA), and partial least squares discriminant analysis (PLS-DA), were conducted using the *metaX* software package (Wen et al., 2017). Variable importance in projection (VIP) scores were calculated, and differential metabolites were identified based on the criteria of VIP > 1 and *P* < 0.05, as determined by *t*-test and fold change (FC) analysis. The identified metabolites and related metabolic pathways were annotated using the KEGG,^1^ HMDB,^2^ and LIPIDMaps^3^ databases.

#### 2.4.7 Correlation analysis of rumen microbiota with serum metabolites and fecal microbiota

Based on the criteria of VIP > 1 and *P* < 0.05, the top 30 serum metabolites identified among the treatment groups, along with the top 10 rumen bacterial genera, were selected for the analysis of changes in metabolic processes. Additionally, the correlations between the top 10 rumen genera and the top 10 fecal genera were examined. Spearman correlation coefficients were calculated using the psych package in R, and significant correlations were visualized using the corrplot package in R.

## 3 Data statistics and analysis

All data were initially organized and subjected to preliminary statistics analysis using Excel 2016. Parameters including growth performance, apparent nutrient digestibility, serum biochemical indices, serum hormone levels, rumen fermentation parameters, and microbial composition were analyzed across treatment groups using SPSS 27.0 (IBM Corp., Armonk, NY, United States). Prior to analysis, data were tested for normality. Data conforming to a normal distribution were analyzed using one-way analysis of variance, and multiple comparisons were performed using Duncan’s method. Results are presented as mean ± standard deviation, with *P* < 0.05 considered statistically significant. Visualization of microbial composition differences was performed using GraphPad Prism version 9.5.1 (GraphPad Software, San Diego, CA, United States).

## 4 Results

### 4.1 Growth performance and apparent digestibilities of dietary nutrients

The ADG in the ME and HE groups was significantly higher than that observed in the LE group, while the FCR in the LE group was significantly higher than in the ME and HE groups (*P* < 0.05). However, no significant differences in ADG or FCR were detected between the ME and HE groups (Table 2). With increasing dietary energy levels, the apparent digestibility of DM, OM, CP, NDF, EE, Ca, and P significantly increased (*P* < 0.05). The apparent digestibility of ADF and CF in the ME and HE groups was also significantly higher than that in the LE group (*P* < 0.05) (Table 3).

**TABLE 2:** The growth performance of yaks receiving rations with different energy levels.

	Dietary NEg levels, MJ/kg		
Item	LE: 4.06	ME: 4.46	HE: 4.87
IBW, kg	247.01 ± 28.51	249.71 ± 44.91	250.56 ± 21.80
FBW, kg	281.75 ± 29.04	303.78 ± 47.47	303.91 ± 30.37
ADG, kg/d	0.75 ± 0.09^b^	1.11 ± 0.31^a^	1.07 ± 0.14^a^
DMI, kg/d	7.35 ± 0.76	7.38 ± 0.83	7.56 ± 0.94
FCR	9.87 ± 1.69^a^	7.01 ± 1.81^b^	7.04 ± 1.50^b^

NEg, net energy for gain; LE, low energy group; ME, medium energy group; HE, high energy group; IBW, initial body weight; FBW, final body weight; ADG, average daily gain; DMI, dry matter intake; FCR, feed conversion rate;

^a, b^Means with different superscript letters in the same column within an item are significantly different from each other (*P* < 0.05).

**TABLE 3:** The apparent digestibility of dietary nutrients in yaks receiving rations with different energy levels.

	Dietary NEg levels, MJ/kg		
Item (%)	LE: 4.06	ME: 4.46	HE: 4.87
DM	66.96 ± 1.52^c^	76.58 ± 1.73^b^	84.19 ± 1.72^a^
OM	68.69 ± 1.57^c^	78.30 ± 1.67^b^	85.08 ± 1.35^a^
CP	60.32 ± 2.40^c^	72.16 ± 2.22^b^	77.41 ± 3.70^a^
CF	55.94 ± 4.61^b^	72.96 ± 1.07^a^	72.69 ± 1.56^a^
NDF	45.13 ± 2.09^c^	51.18 ± 2.81^b^	60.99 ± 3.08^a^
ADF	37.19 ± 1.20^b^	39.43 ± 1.28^a^	40.26 ± 1.45^a^
EE	52.56 ± 7.49^c^	67.84 ± 8.68^b^	76.81 ± 5.49^a^
Ca	39.3 ± 2.65^c^	56.15 ± 3.68^b^	66.87 ± 4.52^a^
P	68.42 ± 4.44^b^	69.79 ± 4.53^b^	77.92 ± 5.60^a^

NEg, net energy for gain; LE, low energy group; ME, medium energy group; HE, high energy group; CP, crude protein; DM, dry matter; NDF, neutral detergent fiber; ADF, acid detergent fiber; EE, ether extract; OM, organic matter; CF, crude fiber; Ca, calcium; P, phosphorus.

^a, b, c^Means with different superscript letters in the same column within an item are significantly different from each other (*P* < 0.05).

### 4.2 Rumen fermentation, serum biochemistry and energy-related hormones

The concentration of MCP increased with rising dietary energy levels, while the concentration of NH_3_-N decreased. Specifically, the MCP concentration in the HE group was significantly higher, and the NH_3_-N concentrations was significantly lower than those in the LE group (*P* < 0.05) (Table 4). The concentrations of GLU, TG, and ALP in the ME and HE groups were significantly higher than those in the LE group (*P* < 0.05). Conversely, the NEFA concentration in the LE group was significantly higher than in the ME and HE groups (*P* < 0.05) (Table 5). Among serum hormones, the GH concentration increased with higher dietary energy levels. Additionally, the concentration of INS, IGF-1, LEP, and T4 in the ME and HE groups were significantly higher than those in the LE group (*P* < 0.05) (Table 6). No significant differences were observed among the groups for the remaining measured indicators.

**TABLE 4:** Rumen fermentation parameters in yaks receiving rations with different energy levels.

	Dietary NEg levels, MJ/kg		
Item	LE: 4.06	ME: 4.46	HE: 4.87
pH	7.27 ± 0.02	7.28 ± 0.08	7.18 ± 0.14
MCP, mg/dL	1.97 ± 0.60^b^	2.05 ± 0.44^b^	2.76 ± 0.32^a^
NH_3_-N, mg/dL	8.35 ± 0.89^a^	7.67 ± 0.73^ab^	7.41 ± 0.50^b^
TVFA, mmol/L	99.62 ± 5.48	99.56 ± 6.61	99.63 ± 5.84
Acetate, mmol/L	68.77 ± 3.62	65.36 ± 5.97	64.35 ± 2.16
Propionate, mmol/L	23.81 ± 2.72	25.25 ± 3.77	27.43 ± 4.49
Butyric acid, mmol/L	8.18 ± 0.72	8.95 ± 0.83	7.85 ± 0.89
Acetate/Propionate	2.92 ± 0.37	2.65 ± 0.55	2.39 ± 0.32

NEg, net energy for gain; LE, low energy group; ME, medium energy group; HE, high energy group; MCP, microbial protein; NH_3_-N, ammonium nitrogen; TVFA, total volatile fatty acids.

^a, b^Means with different superscript letters in the same column within an item are significantly different from each other (*P* < 0.05).

**TABLE 5:** Serum biochemistry of yaks receives rations with different energy levels.

	Dietary NEg levels, MJ/kg		
Item	LE: 4.06	ME: 4.46	HE: 4.87
GLU, mmol/L	3.59 ± 0.54^c^	3.97 ± 0.29^ab^	4.50 ± 0.46^a^
TC, mmol/L	2.22 ± 0.25	2.33 ± 0.30	2.27 ± 0.19
TG, mmol/L	0.22 ± 0.02^c^	0.28 ± 0.07^ab^	0.32 ± 0.10^a^
GLB, g/L	43.88 ± 4.60	41.93 ± 4.86	42.56 ± 5.72
ALB, g/L	30.25 ± 2.27	32.14 ± 1.45	32.33 ± 1.80
TP, g/L	74.13 ± 4.12	74.07 ± 4.60	74.89 ± 4.90
UN, mmol/L	3.60 ± 0.55	3.77 ± 0.43	4.02 ± 0.44
ALT, U/L	41.89 ± 3.34	41.26 ± 4.23	41.72 ± 3.85
AST, U/L	80.76 ± 6.28	81.40 ± 7.97	80.73 ± 6.00
ALP, U/L	157.00 ± 22.07^c^	175.00 ± 11.61^ab^	189.00 ± 23.66^a^
LDH, U/L	1177.32 ± 87.28	1102.00 ± 91.08	1108.38 ± 97.32
NEFA, mmol/L	0.56 ± 0.15^a^	0.50 ± 0.17^ab^	0.38 ± 0.09^c^
β-HB, mmol/L	0.140 ± 0.008	0.138 ± 0.010	0.141 ± 0.011

NEg, net energy for gain; LE, low energy group; ME, medium energy group; HE, high energy group; GLU, glucose; TC, cholesterol; TG, triglyceride; GLB, globulin; ALB, albumin; TP, total protein; UN, urea nitrogen; ALT, alanine aminotransferase; AST, aspartate transaminase; ALP, alkaline phosphatase; LDH, lactate dehydrogenase; β-HB, β-hydroxybutyrate; NEFA, non-esterified fatty acids.

^a, b, c^Means with different superscript letters in the same column within an item are significantly different from each other (*P* < 0.05).

**TABLE 6:** Serum energy-related hormones receive rations with different energy levels.

	Dietary NEg levels, MJ/kg		
Item	LE: 4.06	ME: 4.46	HE: 4.87
GH, ng/mL	12.80 ± 1.22^b^	13.12 ± 1.79^b^	15.06 ± 1.63^a^
GC, pg/mL	91.26 ± 20.26	103.11 ± 24.82	97.13 ± 15.34
IGF-1, ng/mL	19.50 ± 4.63^c^	22.68 ± 4.72^ab^	25.39 ± 5.74^a^
T4, nmol/l	1.35 ± 0.11^b^	1.60 ± 0.17^a^	1.53 ± 0.26^a^
INS, MIU/L	10.61 ± 0.91^b^	12.81 ± 1.19^a^	13.13 ± 1.36^a^
LEP, pg/mL	2.67 ± 0.40^b^	3.51 ± 0.58^a^	3.92 ± 0.73^a^
NE, ng/mL	71.31 ± 3.67	73.36 ± 3.69	74.98 ± 2.68

NEg, net energy for gain; LE, low energy group; ME, medium energy group; HE, high energy group; GH, growth hormone; GC, glucagon; IGF-1, insulin-like growth factor 1; T4, thyroxine; INS, insulin; LEP, leptin; NE, noradrenaline.

^a, b, c^Means with different superscript letters in the same column within an item are significantly different (*P* < 0.05).

### 4.3 Ruminal and fecal 16S rDNA sequencing analysis

**FIGURE 1:**
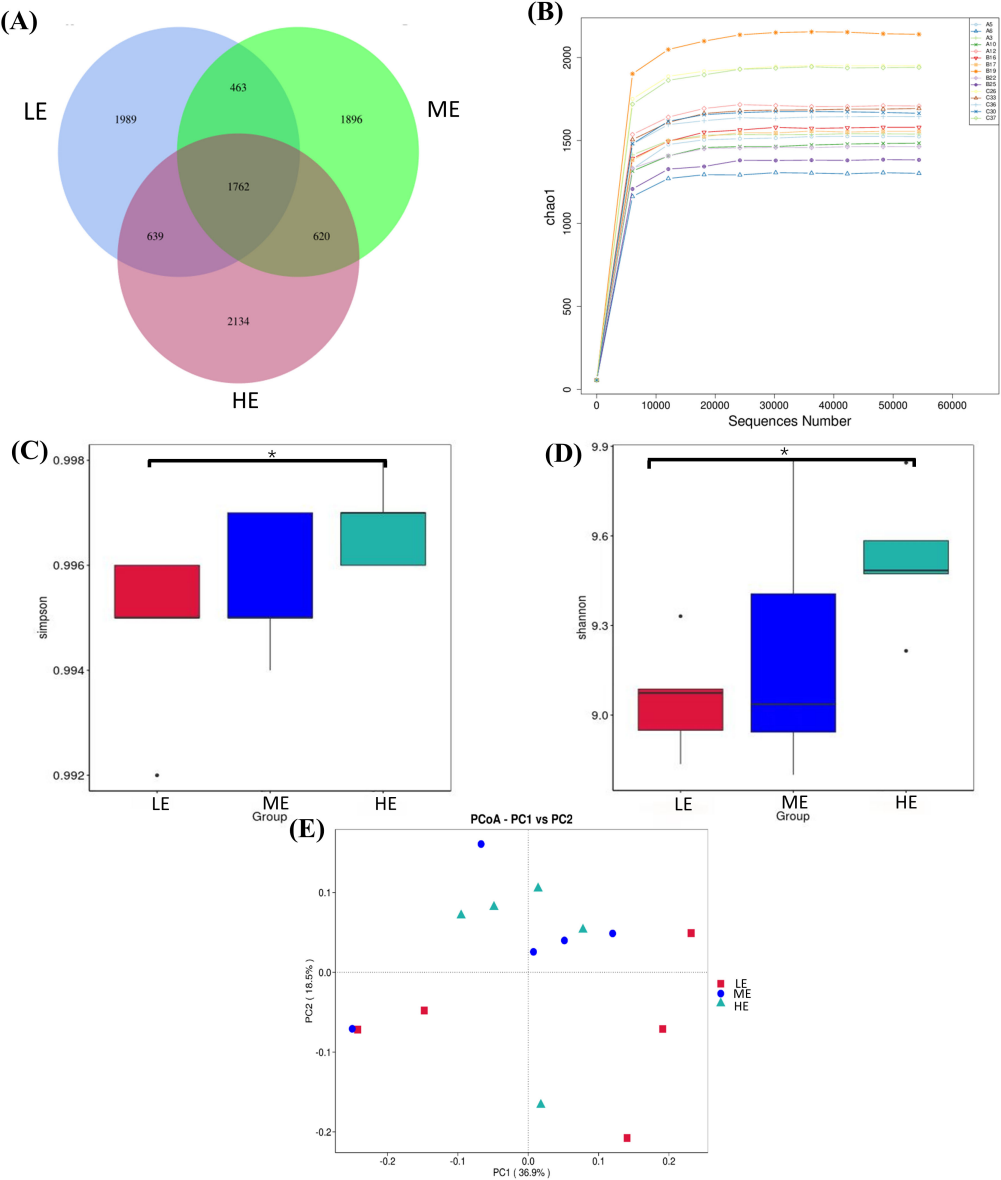
ASVs plot, species rarefaction curve, alpha and beta diversity in the rumen. Venn diagram of ASV in rumen **(A)**. Rarefaction curve of species in rumen **(B)**. Simpson **(C)** and Shannon **(D)** indices of rumen. PCoA analysis in rumen **(E)**. LE, low energy group; ME, medium energy group; HE, high energy group. *Means with different superscript letters in the figure within an item are significantly different (P < 0.05).

**FIGURE 2:**
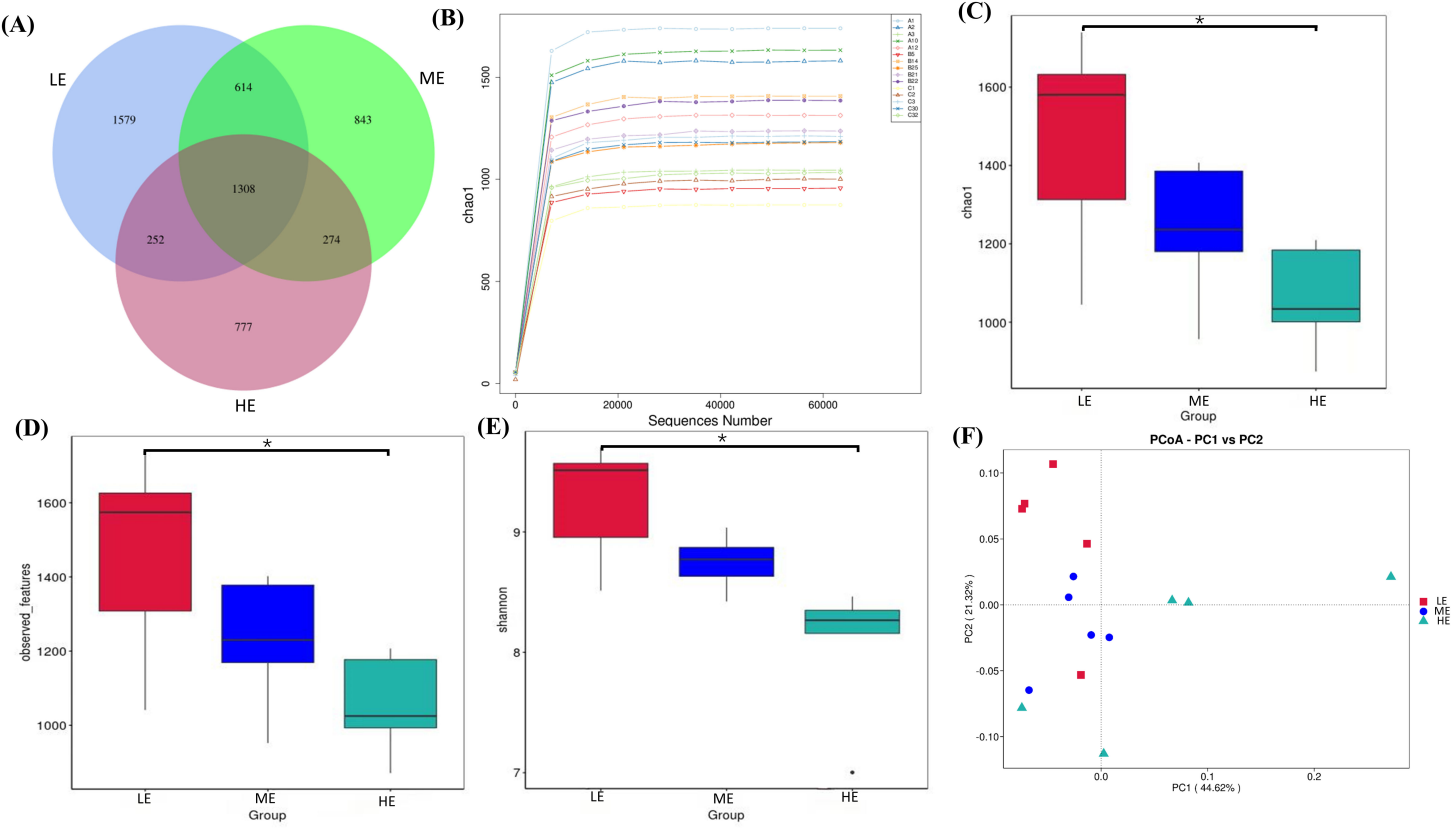
ASVs plot, species rarefaction curve, alpha and beta diversity for feces. Venn diagram of ASV for feces **(A)**. Rarefaction curve of species for feces **(B)**. Chao1 values **(C)**, observed features **(D)**, and Shannon **(E)** index of feces. PCoA analysis of feces **(F)**. LE, low energy group; ME, medium energy group; HE, high energy group. *Means with different superscript letters in the figure within an item are significantly different (*P* < 0.05).

**FIGURE 3:**
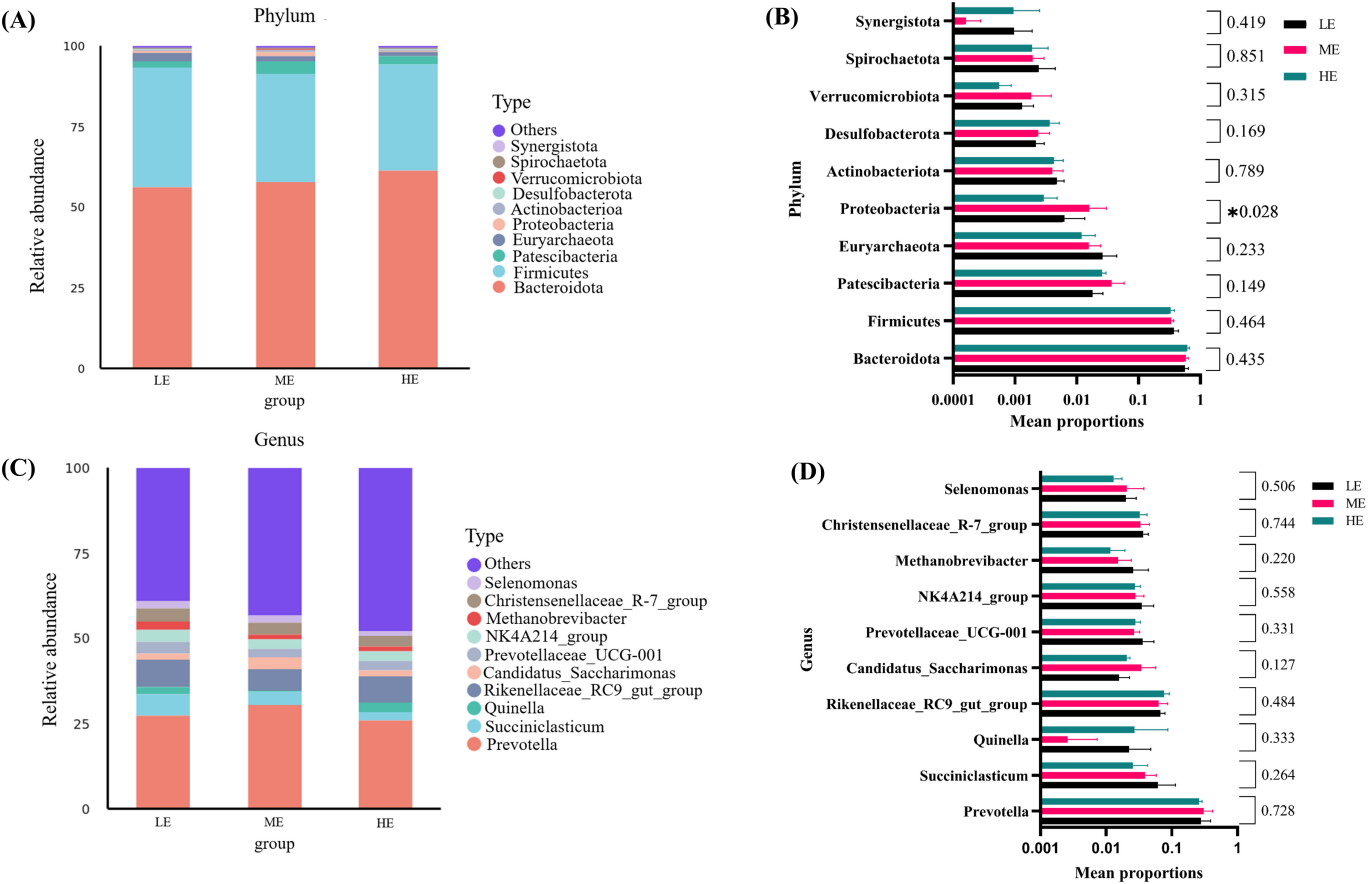
Microbial composition of rumen and differential analysis. Microbial community of rumen abundance at phylum **(A)** and genus **(C)** levels. One-way analysis of variance in the percentage of microbial community of rumen abundance at the phylum **(B)** and genus **(D)** levels. LE, low energy group; ME, medium energy group; HE, high energy group. *Means with different superscript letters in the figure within an item are significantly different (*P* < 0.05).

**FIGURE 4:**
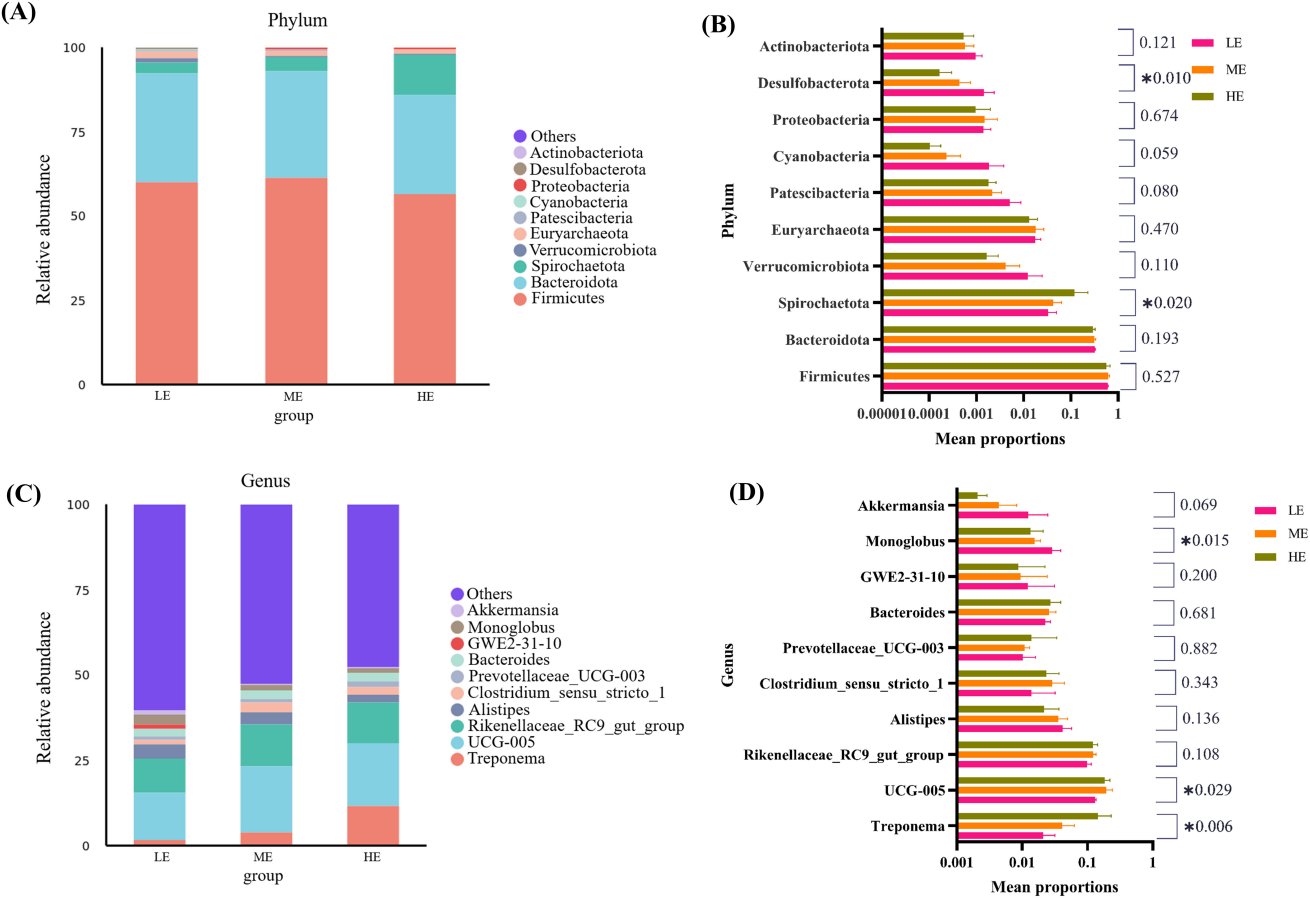
Fecal microbial composition and differential analysis. Fecal microbial community abundance at phylum **(A)** and genus **(C)** levels. One-way analysis of variance in percentage of fecal microbial community abundance at the phylum **(B)** and genus **(D)** levels. LE, low energy group; ME, medium energy group; HE, high energy group. *Means with different superscript letters in the figure within an item are significantly different from each other (*P* < 0.05).

Following 16S rDNA sequencing of rumen and fecal samples, a total of 9,503 and 5,647 valid ASVs were obtained for the rumen and feces, respectively. Specifically, the number of ASVs in the rumen samples form the LE, ME, and HE groups was 4,853, 4,741, and 5,155, respectively (Figure 1A), while the number of ASVs in the fecal samples from the LE, ME, and HE groups was 3,753, 3,039, and 2,611, respectively (Figure 2A). The rarefaction curves of both rumen and fecal samples plateaued with increasing sequencing depth, indicating that the sequencing depth was sufficient for capturing the majority of microbial diversity (Figures 1B, 2B). In the rumen samples, both the Simpson and Shannon diversity indices increased with rising dietary energy levels, with values in the LE group significantly lower than those in the HE group (*P* < 0.05) (Figures 1C,D; Table 7). Conversely, in the fecal samples, the Shannon, Chao1, and observed features indices decreased as the dietary energy level increased, with these indices significantly lower in the HE group compared to the LE group (*P* < 0.05) (Figures 2C–E; Table 8). PCoA demonstrated clear separations among the LE, ME, and HE groups in both rumen and fecal microbiota compositions (Figures 1E, 2F). At the phylum level, *Firmicutes* and *Bacteroidetes* were the dominant phyla in the rumen microbiota across all groups. Notably, the relative abundance of *Proteobacteria* was significantly higher in the ME group compared to the LE and HE groups (*P* < 0.05) (Figures 3A,B). At the genus level, *Prevotella* was the predominant genus across all groups, with a higher relative abundance observed in the ME group. In addition, the relative abundance of *Prevotellaceae UCG-001* was higher in the LE group compared to the ME and HE groups (Figures 3C,D). In the fecal microbiota, *Firmicutes*, and *Bacteroidetes* remained the dominant phyla across groups. The relative abundance of *Spirochaetota* was significantly higher in the HE group than in the LE and ME groups, while the relative abundance of *Desulfobacterota* was significantly lower in the HE group compared to the LE and ME groups (*P* < 0.05) (Figures 4A,B). At the genus level, the relative abundance of *Treponema* increased significantly with rising dietary energy levels (*P* < 0.05). Additionally, the relative abundance of *Monoglobus* was significantly higher in the LE group than in the ME and HE groups, whereas the relative abundance of *UCG-005* was significantly lower in the LE group compared to the ME and HE groups (*P* < 0.05) (Figures 4C,D).

**TABLE 7:** Effects of diets with different energy levels on the rumen alpha diversity index of yaks.

	Dietary NEg levels, MJ/kg		
Item	LE: 4.06	ME: 4.46	HE: 4.87
Chao1	1511.45 ± 145.21	1624.22 ± 299.01	1778.68 ± 153.62
Shannon	9.06 ± 0.18^b^	9.21 ± 0.42^ab^	9.52 ± 0.23^a^
Simpson	0.995 ± 0.002^b^	0.996 ± 0.001^ab^	0.997 ± 0.008^a^
Observed features	1501.80 ± 144.57	1613.40 ± 300.87	1768.80 ± 152.00

NEg, net energy for gain; LE, low energy group; ME, medium energy group; HE, high energy group.

^a, b^Means with different superscript letters in the same column within an item are significantly different from each other (*P* < 0.05).

**TABLE 8:** Effects of different energy level diets on the alpha diversity index of yak feces.

	Dietary NEg levels, MJ/kg		
Item	LE: 4.06	ME: 4.46	HE: 4.87
Chao1	1462.07 ± 281.31^a^	1233.15 ± 182.13^ab^	1060.58 ± 138.39^b^
Shannon	9.25 ± 0.50^a^	8.75 ± 0.23^ab^	8.05 ± 0.59^b^
Simpson	0.996 ± 0.001	0.994 ± 0.001	0.974 ± 0.034
Observed features	1457.00 ± 287.78^a^	1226.20 ± 181.95^ab^	1054.60 ± 138.37^b^

NEg, net energy for gain; LE, low energy group; ME, medium energy group; HE, high energy group.

^a, b^Means with different superscript letters in the same column within an item are significantly different from each other (*P* < 0.05).

### 4.4 Screening of serum differential metabolites and pathway analysis

**FIGURE 5:**
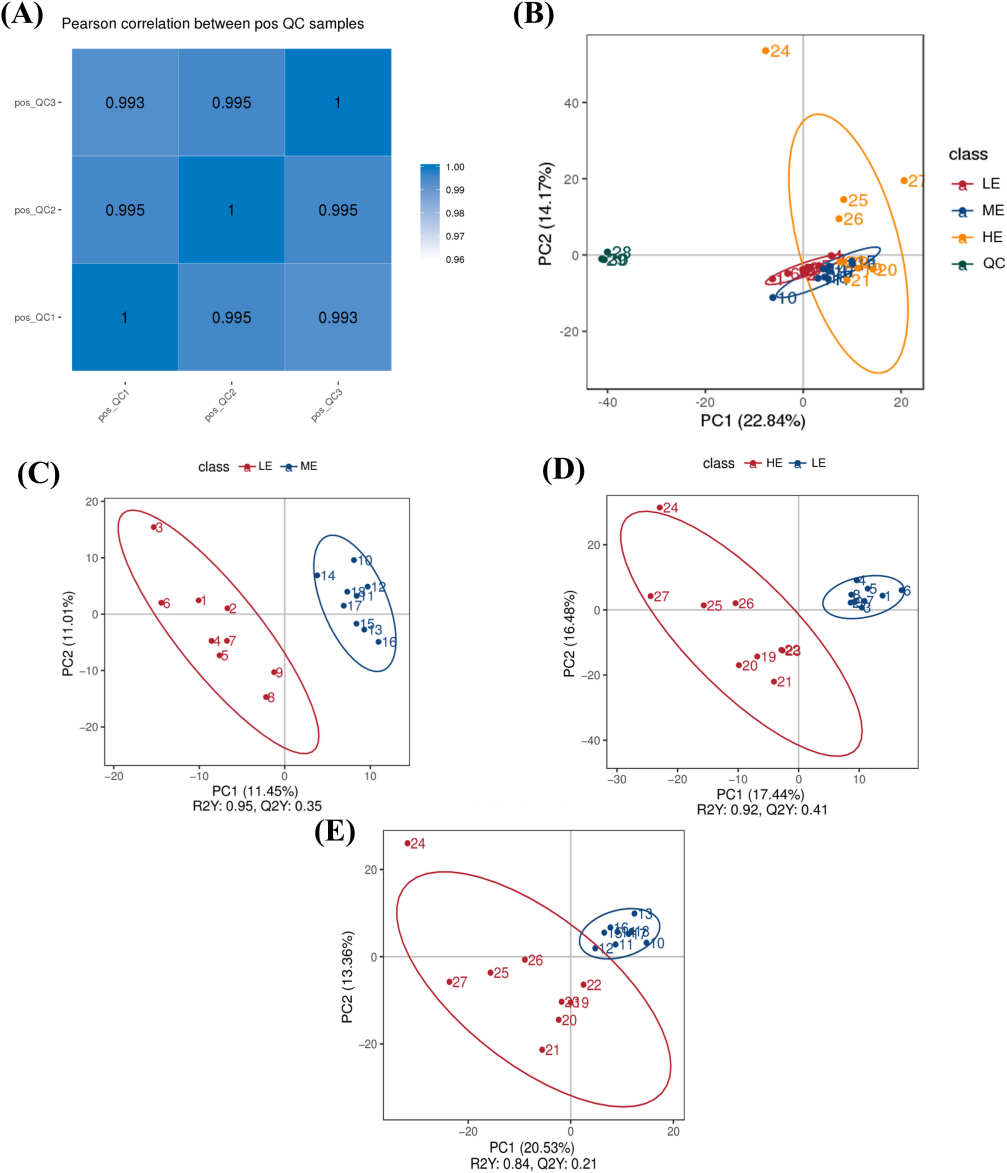
Identification, quantification, and statistical comparison of serum LC-MC metabolites. Pearson’s correlation coefficient between QC samples **(A)**. PCA analysis of serum **(B)**. PLS-DA analysis of serum **(C–E)**. PC1 is the first principal component. PC2 is the second principal component. LE, low energy group; ME, medium energy group; HE, high energy group.

The Pearson correlation coefficients among all datasets exceeded 0.99 (Figure 5A), indicating strong stability and high data quality throughout the detection process. PCA score plots demonstrated good clustering within groups and clear separation between groups across the three dietary treatments, confirming the validity of the experimental data (Figure 5B). The PLS-DA plot showed that the samples were distributed within the Hoteling T^2^ ellipse range, and permutation tests between groups (LE vs. ME, R^2^Y = 0.95, Q^2^Y = 0.35; LE vs. HE, R^2^Y = 0.92, Q^2^Y = 0.41; ME vs. HE, R^2^Y = 0.84, Q^2^Y = 0.21) further confirmed the stability, reliability and validity of the model (Figures 5C–E).

**FIGURE 6:**
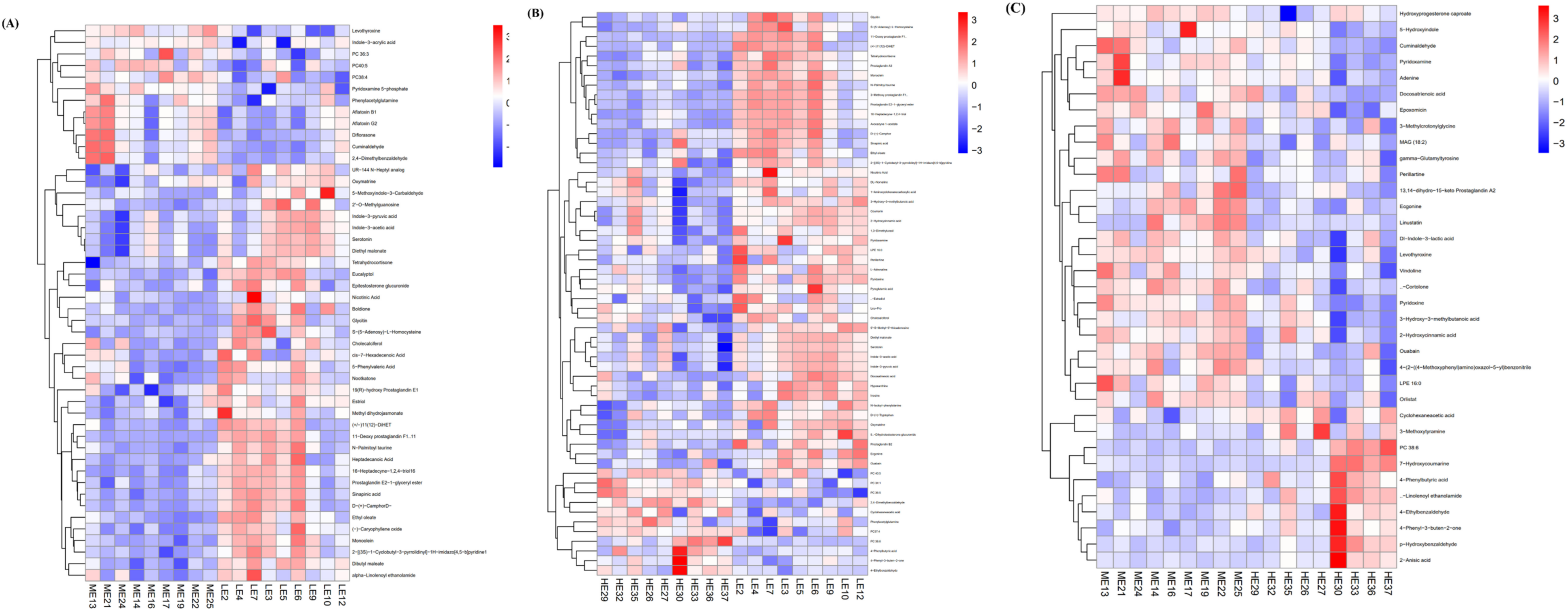
Clustering heatmaps of differential metabolites. Heatmaps of differential serum metabolites of LE vs ME **(A)**, LE vs HE **(B)**, and ME vs. HE **(C)**. The tree diagram of metabolites cluster on the left side shows that each column shows a sample, the lower side shows the name of the sample, and each row shows a metabolite. LE, low energy group; ME, middle energy group; HE, high energy group.

Metabolic differences between groups were screened using criteria of *P* < 0.05 and VIP > 1. A total of 48 differential metabolites were identified between the LE and ME groups, including phosphatidylcholine 36:3 (PC 36:3), phosphatidylcholine 40:5 (PC 40:5), phosphatidylcholine 38:4 (PC 38:4), indole-3-acrylic acid, and pyridoxamine 5-phosphate, among others (Figure 6A). In the comparison between the LE and HE groups, 58 differential metabolites were detected, with significant metabolites including phosphatidylcholine 34:1 (PC 34:1), phosphatidylcholine 38:6 (PC 38:6), phosphatidylcholine 40:5 (PC 40:5), pyridoxine, and pyridoxamine (Figure 6B). Additionally, 35 differential metabolites were identified between the ME and HE groups, with key metabolites including phosphatidylcholine 38:6 (PC 38:6), DL-indole-3-lactic acid, pyridoxamine, pyridoxine, and adenine (Figure 6C).

**FIGURE 7:**
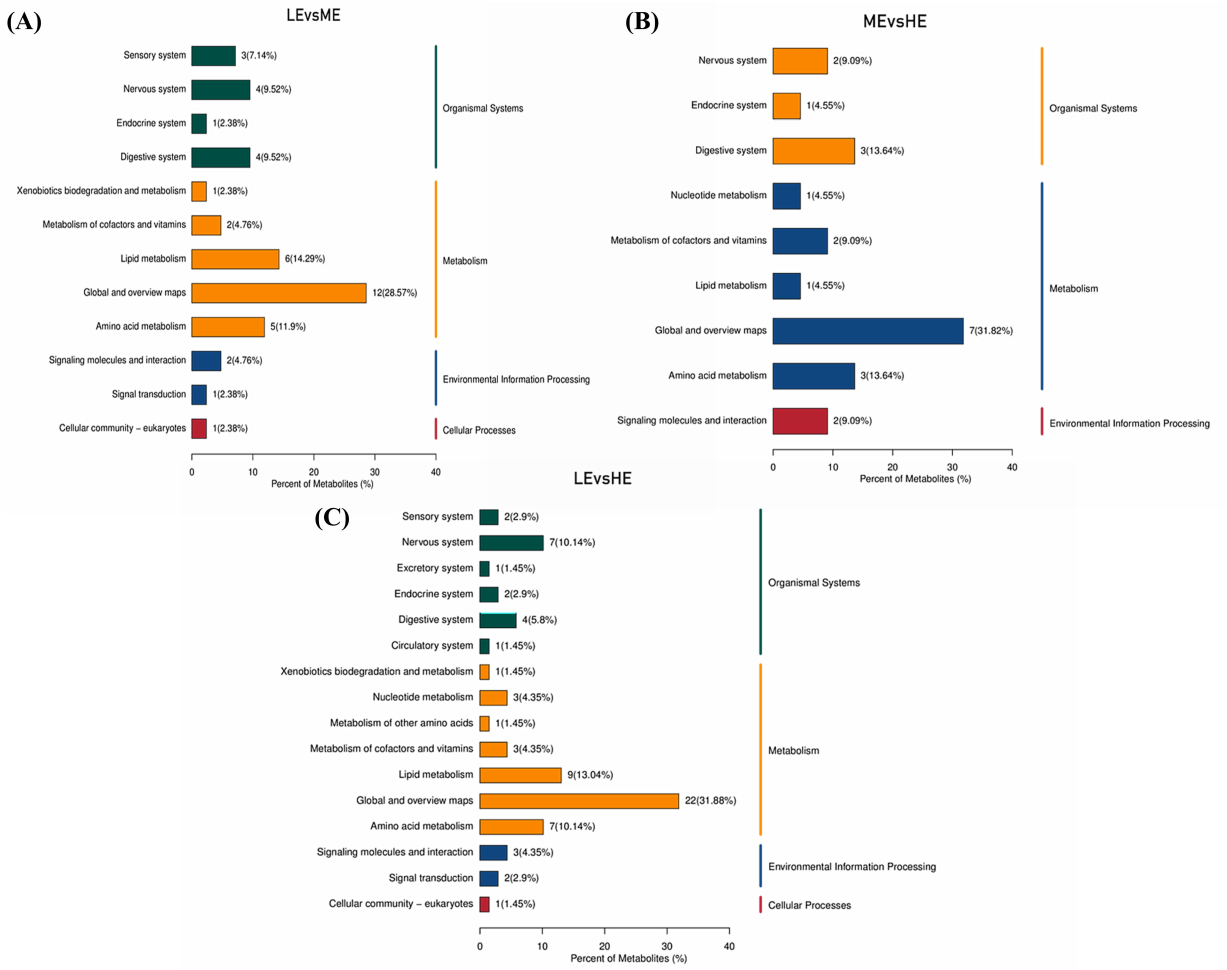
KEGG pathway enrichment map of differential metabolites. The different metabolic pathways of LE vs. ME **(A)**, LE vs. HE **(B)**, and ME vs. HE **(C)** in the KEGG database. The *x-*axis in the figure represents the percentage of metabolites annotated under a specific KEGG pathway relative to the total number of annotated metabolites, while the right side of the *y-*axis shows the primary classification of KEGG pathways, and the left side shows the secondary classification of KEGG pathways. LE, low energy group; ME, medium energy group; HE, high energy group.

Enrichment analysis of the differential metabolites using the KEGG database, based on an impact coefficient (Impact > 0.1) and a *P* < 0.05, revealed that these serum differential metabolites across the three groups were primarily enriched in pathways related to amino acid metabolism, vitamin metabolism, nucleotide metabolism, lipid metabolism, and other secondary metabolite pathways (Figures 7A–C).

### 4.5 Correlation analysis between rumen microbiota and serum metabolites, and between rumen and fecal microbiota

**FIGURE 8:**
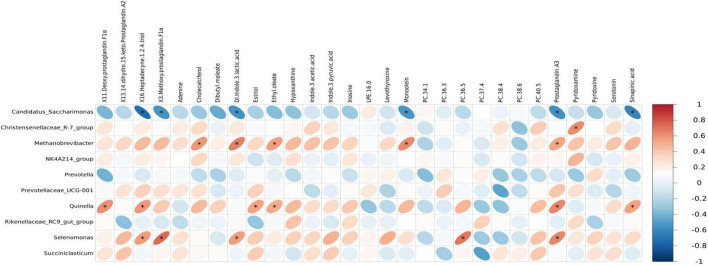
Heatmap of correlation between rumen microbiota and serum differential metabolites. Each row in the figure represents a metabolite, each column represents a microbiota at the genus level, and each lattice represents the Spearman correlation coefficient between a metabolite and microbiota at the genus level. Red color indicates positive correlation, while blue color indicates negative correlation. asterisk (*) mark indicates statistical significance, that is, *P* < 0.05.

**FIGURE 9:**
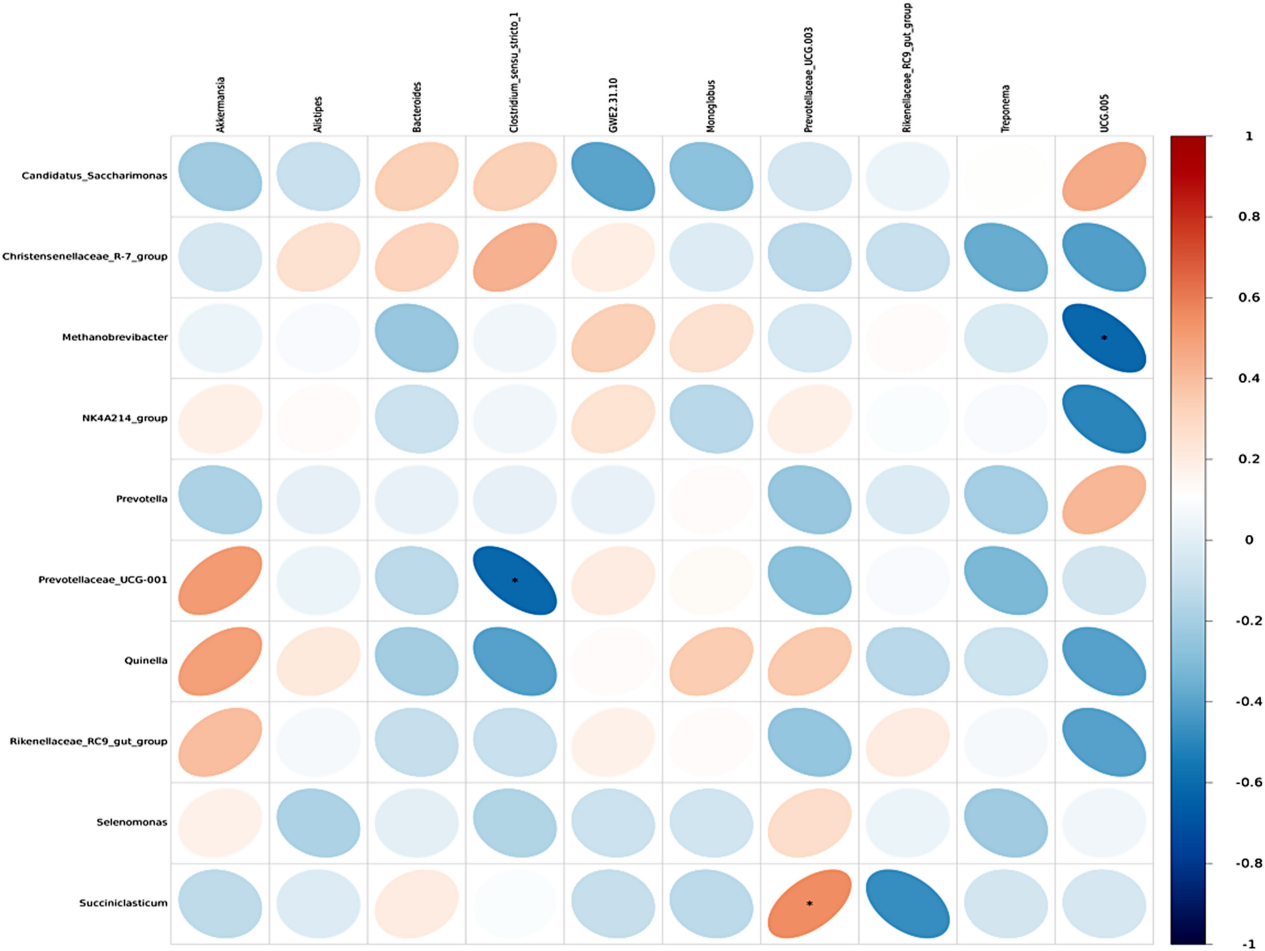
Heatmap of correlation between fecal microbiota and rumen microbiota. Each row in the figure represents a fecal microbiota at genus level, each column represents a rumen microbiota at genus level, and each lattice represents the Spearman correlation coefficient between rumen and fecal microbiota at genus level. Red for positive correlation, while blue for negative correlation. asterisk (*) mark indicates statistical significance, that is *P* < 0.05.

Spearman correlation analysis was conducted to examine the associations between rumen microbiota and serum metabolites, as well as between rumen and fecal microbiota, with the results visualized through correlation heatmaps. Initially, the top 30 differential serum metabolites and the top 10 rumen bacterial genera across the three dietary groups were selected for analysis. The results revealed that the genera *Quinella, Selenomonas, Methanobrevibacter*, and *Christensenellacece R-7 group* were positively correlated with multiple serum differential metabolites, showing positive associations with six, five, five, and one serum metabolites, respectively. In contrast, *Candidatus Saccharimonas* exhibited negative correlations with six serum differential metabolites (Figure 8). Overall, the serum differential metabolites correlated with *Quinella, Selenomonas*, and *Methanobrevibacter* were primarily associated with lipid metabolism pathways. Further analysis was performed on the top 10 dominant genera in the rumen and feces across the three groups to investigate the interrelationships between rumen and fecal microbiota. The results demonstrated specific correlations between microbial genera in the rumen and those in the feces. Notably, *Prevotellaceae UCG-001* in the rumen was negatively correlated with *Clostridium sensu stricto*-1 in the feces. while *Succiniclasticum* in the rumen showed a positive correlation with *Prevotellaceae UCG-003* in the feces. Additionally, *Methanobrevibacter* in the rumen exhibited a negative correlation with *UCG-005* in the feces (Figure 9).

## 5 Discussion

### 5.1 Effect of dietary energy on average daily gain and nutrient digestion

Genetics, nutrition, and feeding management are key factors influencing yak production. Compared with traditional grazing system, house-feeding and supplementary feeding have been shown to significantly improve yak growth performance (Hu et al., 2021; Dai et al., 2022). Among nutritional strategies, modulating dietary energy and protein levels remains a common and effective approach to enhance growth performance (Sun et al., 2015a). Research on the energy and protein requirements for yaks has gradually increased, with the Indian Council of Agricultural Research (Indian Council of Agricultural Research, 2013) providing guidelines for the nutrient requirements of Yak and Mithun. Additionally, the Qinghai Yak Engineering Technology Research Center has developed a metabolic energy model for yaks at different physiological stages (Hu, 1997; Hao et al., 2020). Studies have also investigated the dietary energy requirements of various yak breeds, including *Tianzhubai* yak (Liu, 2022)*, Datong yak* (Yao et al., 2022)*, Maiwa* yak (Wang et al., 2017), and *Niangya* yak (Jiang et al., 2024). However, due to differences in breeds and regional conditions, a unified nutritional standard for yaks has yet to be established. In a study by Yao et al. (2022), three dietary NEg levels (5.51 MJ/kg with 16.72% CP, 6.22 MJ/kg with 16.96% CP, 6.94 MJ/kg with 17.27% CP) were evaluated, and it was found that the ADG increased by 29.69% in the 6.22 MJ/kg group and by 21.88% in the 6.94 MJ/kg group compared with the 5.51 MJ/kg group. Similarly, Ahmad et al. (2020) reported that yaks fed diets with NEg levels of 5.5, 6.2, and 6.9 MJ/kg at a concentrated-to-forage ratio of 30:70 exhibited ADG of 0.63, 0.88, and 0.86 kg/d. respectively. These findings are consistent with the results of the present study, which demonstrated that the ADG in the ME and HE groups was significantly higher than that in the LE group. Similarly, Yang et al. (2013) observed that yak calves fed diets with NEg levels of 6.50 and 7.40 MJ/kg had significantly higher ADG compared with those in the 5.60 MJ/kg group, with the FCR being significantly higher in the 5.60 MJ/kg group than in the other groups.

The digestibility of dietary nutrients plays a crucial role in the growth and development of ruminants (Zhang et al., 2022). Previous studies have shown that increasing dietary energy levels enhances the apparent digestibility of DM, OM, CP, and EE, a finding consistent with research conducted in steers (Navarrete et al., 2017) and cows (Su et al., 2017). The energy-to-protein ratio is an important indicator for evaluating the balance between dietary energy and protein, which directly influences the potential for growth in animals (Medhi et al., 2018; Liu H. et al., 2022). In the present study, the CP level in the diets was kept consistent across groups, and it was observed that increasing dietary energy levels improved the energy-to-protein ratio, thereby supporting enhanced yak growth. Bao et al. (2020) investigated the effects of three net energy (NE) levels (7.64 MJ/kg with 9.82% CP, 8.95 MJ/kg with 10.57% CP, 10.18 MJ/kg with 10.94% CP) in TMR diets for house-fed fattening yaks, reporting that the apparent digestibility of CP, ADF, NDF, and P improved with higher nutritional levels. Similarly, in the present study, higher dietary energy levels were associated with improved apparent digestibility of P, CP, OM, and NDF. However, no significant differences were observed in the apparent digestibility of ADF and CF between the ME and HE groups. Zhu et al. (2023) evaluated metabolizable energy levels of 7.20, 7.89, and 8.58 MJ/kg with 9.0% CP, and found that NDF digestibility was significantly higher in the 7.89 and 8.58 MJ/kg groups compared with the 7.20 MJ/kg group, whereas ADF digestibility did not differ across the groups. This finding suggest that within a certain range of dietary energy levels, further increases in energy intake may not lead to additional improvements in the digestibility of certain nutrients.

### 5.2 Effects of different dietary energy on rumen fermentation parameters, serum biochemical indices and serum energy-related hormones of yaks

The rumen pH value is a critical indicator reflecting the stability of the rumen environment, with a normal range for ruminants typically between 6.2 and 7.2 (Van Soest et al., 1991). In yaks, rumen pH generally ranges from 6.4 to 7.7, which is slightly higher than that observed in other beef cattle (Liu et al., 2019; Pang et al., 2022; Liu X. et al., 2023). In the present study, the pH value ranged from 7.18 to 7.27, consistent with these previous findings. VFAs provide more than 70% of the energy required by ruminants (Zanton and Heinrichs, 2009; Achmadi et al., 2012). The production of acetate is primarily associated with the proportion of roughage in the diet, while the concentration of propionate is linked to the proportion of dietary concentrate (Dórea et al., 2020). In the present study, no significant differences were observed in the concentration of acetic acid and propionic acid among the three groups. However, with increasing dietary energy levels, the concentration of propionic acid increased, while the concentration of acetic acid decreased, which aligns with the findings of Jing et al. (2020) and Chen et al. (2024). This trend may be attributed to the influence of dietary energy levels on the composition and abundance of rumen microbial populations (Li et al., 2019). The optimal concentration of NH_3_-N in the rumen has been reported to range between 6 and 30 mg/dL (Preston et al., 1965). In the current study, the NH_3_-N concentration fell within this optimal range, indicating that microbial growth and activity were not adversely affected. Nitrogen-containing compounds are degraded into ammonia nitrogen in the rumen, which is then utilized by microbes as a nitrogen source for the synthesis of microbial protein (Pisulewski et al., 1981; Zhou et al., 2022). As dietary energy levels increased in this study, the concentration of NH_3_-N decreased, while the content of MCP significantly increased. These results suggest that increasing dietary energy levels enhance the availability of nitrogen for rumen microbes, thereby promoting the conversion of NH_3_-N into MCP and facilitating an improved energy-nitrogen balance (Zhou et al., 2019). Similar trends in NH_3_-N concentrations have also been reported by Wang et al. (2017) in their study on yaks fed with diets containing NEg levels of 4.17, 4.48, and 4.79 MJ/kg and a CP level of 14.81% under house-feeding condition.

Blood physiological parameters, including biochemical indicators and endocrine hormone levels, serve as quantitative indicators of the internal physiological state of an organism and play a critical role in evaluating animal health. Glucose, a primary energy substrate, typically ranges from 3.9 to 5.3 mmol/L in yaks (Liu et al., 2015; Wang, 2015). Previous studies have demonstrated that serum glucose levels in yaks increase with a higher energy-to-nitrogen ratio (Liu H. et al., 2022). In the present study, glucose concentrations in the ME and HE groups were significantly higher than those in the LE group, consistent with the observed trend in ADG. TG contribute to energy regulation by serving as a reservoir for hydrolyzable non-esterified fatty acids (De Koster and Opsomer, 2013). The TG levels was significantly higher in the ME and HE groups compared to the LE group, whereas NEFA levels were correspondingly lower in these groups. ALP, a marker enzyme associated with osteoblast differentiation and bone metabolism (Sheng et al., 2022), exhibited a trend similar to that of TG and glucose. These findings suggest that increasing dietary energy levels can enhance the conversion of NEFA to TG in the blood, thereby improving glucose and lipid metabolism, supporting body weight gain, and promoting energy balance and fat deposition in yaks. The concentration of β-HB are indicative of an animal’s energy status, with elevated concentrations signaling increased ketone body production (Bonfatti et al., 2019). β-HB concentration exceeding 0.8 mmol/L are typically considered indicative of ketosis. In this study, β-HB levels ranged from 0.13 to 0.14 mmol/L, well below the ketosis threshold, suggesting a favorable energy status in all groups (Pichler et al., 2014). ALT and AST are biomarkers for liver function, with elevated levels commonly associated with hepatic damage (Sun et al., 2015b; Liu H. et al., 2022). In the present experiment, ALT and AST levels remained within the normal physiological range, and no significant differences were observed among the treatment groups, indicating that the dietary energy levels employed did not adversely affect liver function (Qiao, 2013). Indicators such as UN, TP, GLB, and ALB reflect protein metabolism and amino acid balance (Qin et al., 2022). No significant differences were observed in these parameters among the dietary groups, likely due to the consistent crude protein content across all experimental diets. The finding is consistent with results reported by Zhu et al. (2023), who found no significant changes in protein metabolism indices in yaks fed diets with metabolizable energy levels of 7.20, 7.89, and 8.58 MJ/kg and identical CP levels.

Hormones play a critical role in regulating energy metabolism, growth, and development in animals. Numerous studies have demonstrated that GH is positively correlated with the ADG of ruminants (Sarkar et al., 2008; Tomczak et al., 2019). It has been reported that dietary energy level influences the growth performance of yaks through the actions of both GH and IGF-1 (Miyake et al., 2016). Specifically, IGF-1 contributes to the enhancement of ADG with increasing dietary energy levels and does not rely solely on GH to mediate growth performance (Yang et al., 2019). In the present study, IGF-1 levels in the ME and HE groups were significantly higher than those in the LE group, while no significant difference in ADG was observed between the ME and HE groups. IGF-1 is also positively correlated with INS, jointly promoting the production performance in beef cattle (Ciccioli et al., 2003; Dance et al., 2017). In this study, both IGF-1 and INS concentrations in the ME and HE groups were significantly higher than those in the LE group, indicating that increasing dietary energy levels appropriately can enhance glucose metabolism in yaks, thereby promoting growth, development, and production performance. This finding is consistent with results reported in Tibetan sheep (Zhou et al., 2019). LEP, secreted by adipocytes, plays a role in inhibiting excessive fat deposition, while the expression of the leptin gene in cattle has been shown to promote weight gain (Nkrumah et al., 2005). In the current experiment, leptin levels in the ME and HE groups were significantly higher than those in the LE group. The increase in dietary energy level likely led to elevated fat deposition in yaks, thereby enhancing leptin secretion. Additionally, Thyroid-stimulating hormone (TSH) promotes the release of leptin, which in turn stimulates the production of T4 (Duntas and Biondi, 2013). This mechanism may explain the significantly higher serum T4 concentrations observed in the ME and HE groups compared to the LE group in this study.

### 5.3 Effects of dietary energy level on rumen and fecal microbiota of yaks

The rumen microbiota plays a critical role in maintaining the health and normal digestive function of ruminants, contributing to the harvesting of 85–90% of the gross energy available from the diet (Ley et al., 2008). In the present study, the Shannon and Simpson indices revealed that microbial diversity was lowest in the LE group, indicating that excessively low dietary energy levels reduce microbial richness (Yang et al., 2021). Similarly, a study examining the NEg levels of 5.5, 6.2, and 6.9 MJ/kg in yak rumen microbiota reported that the Shannon and Simpson indices were higher at 6.2 and 6.9 MJ/kg compared to 5.5 MJ/kg, a trend that consistent with the findings of this study (Ahmad et al., 2020). PCoA analysis revealed that dietary energy level exerted a substantial influence on the composition of rumen microbiota (Islam et al., 2021). *Firmicutes* and *Bacteroidota* were identified as the dominant phyla in the rumen microbiota of yaks, consistent with previous studies (Pang, 2023; Zhang et al., 2023). *Firmicutes* are primarily involved in the degradation of fibrous materials, whereas *Bacteroidetes* mainly degrade non-fibrous materials (Evans et al., 2011; Reigstad and Kashyap, 2013). The present findings indicated that with increasing dietary energy levels, the relative abundance of *Bacteroidetes* increased, while that of *Firmicutes* decreased, although no significant differences were observed among the groups. This contrasts with some previous research results, which reported significant variations in these phyla with dietary energy level adjustments (Zhou et al., 2017; Liu et al., 2019). *Proteobacteria* play an important role in biofilm formation, fermentation, and the digestion of soluble carbohydrates (Pitta et al., 2016). Previous research in dairy cows has shown that the abundance of *Proteobacteria* increases with high-concentrate diets (Kibegwa et al., 2023). In the current study, the abundance of *Proteobacteria* was significantly highest in the ME group. It is hypothesized that the rumen environment developed under the ME diet represents a threshold for a linear increase in *Proteobacteria* abundance, thereby creating favorable conditions for efficient rumen fermentation. At the genus level, the relative abundance of *Prevotella* was highest in the ME group, consistent with findings in yak by Ma et al. (2023). *Prevotella* is essential for protein and starch metabolism and is capable of utilizing pectin and cellulose, contributing to improved feed utilization efficiency (Bekele et al., 2010). These findings suggest that the energy level of the ME diet in this study may be optimal for enhancing dietary utilization efficiency in yaks. *Prevotellaceae UCG-001* has been reported to promote the resynthesis of branched-chain fatty acids through the elongation of valeric acid and propionic acid (Bainbridge et al., 2018). In this study, the lowest concentration of propionic acid in the rumen was found in the LE group, which exhibited the highest abundance of *Prevotellaceae UCG-001.* This may indicate that *Prevotellaceae UCG-001* utilized propionic acid for the synthesis of other branched-chain fatty acids under low-energy diets, thereby influencing the rumen volatile fatty acid profile in yaks.

Fermentation in the hindgut contributes approximately 10–15% of the gross energy available to adult cattle, underscoring the critical role of hindgut bacteria in energy extraction and growth in ruminants (Fan et al., 2019). In dairy cows, high-concentrate diets have been reported to remodel the composition and function of hindgut microbiota (Zhang et al., 2021). In the present study, the alpha diversity indices, including observed features, Chao1, and Shannon, were significantly higher in the LE group compared to the HE group. Beta diversity analyses demonstrated clear differences in microbial community distribution among the three groups, indicating that dietary energy levels exert a substantial influence on the composition of fecal microbiota. These findings suggest that excessive dietary energy may inhibit the proliferation of fecal microbiota. *Firmicutes* and *Bacteroidota* accounted for over 90% of the fecal microbiota, consistent with observations in beef cattle (Zhang et al., 2023) and Holstein calves (Gao, 2018). The phylum *Spirochaetota* has been associated with the energy metabolism of ruminants, with its abundance increasing alongside elevated dietary energy levels (Bi et al., 2018; Pal et al., 2021). In this study, the abundance of *Spirochaetota* significantly increases with higher dietary energy levels, indicating that raising the dietary energy may enhance energy metabolism in the hindgut. *Desulfobacterota* contributes to creating an anaerobic environment favorable for gastrointestinal fermentation (Cai, 2021). The lowest abundance of *Desulfobacterota* was observed in the HE group, indicating that excessively high dietary energy levels may negatively affect hindgut fermentation conditions. Additionally, the abundance of *Treponema*, a genus within the phylum *Spirochaetota*, increased significantly with higher dietary energy levels. *Treponema* plays a beneficial role in nutrient metabolism, consistent with findings in yaks reported by Shen et al. (2024). The relative abundance of *UCG-005* was highest in the ME group in this study. A previous study in goats indicated that the abundance of *UCG-005* increased with higher NDF levels (Zhang and Wang, 2018). *Monoglobus*, a genus within *Firmicutes* known for its role in degrading pectin and fiber substances (Kim et al., 2019), exhibited significantly lower abundance in the HE groups compared to the LE group. This aligns with the current findings, suggesting that while appropriate increases in dietary energy support the growth of fiber-degrading bacteria, excessive energy levels may exert inhibitory effects. These results collectively indicate that the selection of dietary energy levels should be approached from a comprehensive perspective, considering both energy metabolism and hindgut microbial balance to align with specific feeding objectives in yak production systems.

### 5.4 Effects of dietary energy levels on serum metabolites and metabolic pathways

Metabolomics data provide valuable insights into the physiological and metabolic status of animals (Vinayavekhin et al., 2010). Phosphatidylcholine (PC) acts as a storage molecule for choline, with its metabolites participating in the glycerophospholipid metabolism pathway (Mohamad et al., 2015). Choline supplementation has been shown to aid in preventing fatty liver in early lactating dairy cows (Lu et al., 2023; Lima et al., 2024). In the present study, the levels of phosphatidylcholine substances (PC 40:5, 34:1, 38:6) were significantly upregulated in the HE group compared to LE and ME groups. This suggests that a high-energy diet may influence lipid metabolism by altering lipid levels and their metabolites, thereby increasing the availability of substrates for energy metabolism in the liver and peripheral tissues (Bäckhed and Crawford, 2010). Pyridoxine, pyridoxamine, and pyridoxamine 5-phosphate present the active forms of vitamin B6, which are essential for efficient protein synthesis and critical for growth, lactation, and overall health in animals (Magan et al., 2020; Wu et al., 2022). In this study, the content of active vitamin B6 was highest in the ME group, indicating that this dietary energy level may be more suitable for supporting the growth and development of yaks. DL-indole-3-lactic acid and indole-3-acrylic acid are key metabolites of the tryptophan metabolic pathway. As a functional essential amino acid, tryptophan plays a significant role in influencing growth performance, feed intake, lactation, and the body’s antioxidant and immune functions (Li et al., 2016). The results demonstrated that the content of indole-3-acrylic in the ME group was higher than that in the LE group, while the level of DL-indole-3-lactic acid in the ME group exceeded that observed in the HE group. This finding indicates that appropriate increases in dietary energy levels can support improved growth performance and feed intake in yaks. Adenine is integral to energy metabolism and is involved in the synthesis of DNA and RNA within cells. Supplementation with nucleotides in the diets of piglets has been reported to enhance growth performance and immunity (Diehl et al., 2022). In the current study, adenine was found to be highly expressed in the ME group, suggesting that a medium energy diet may facilitate the promotion of growth and enhance cellular energy metabolism in yaks.

### 5.5 Correlation analysis between rumen microbiota, serum metabolomics, and fecal microbiota

Quinella exhibited a positive correlation with several differential serum metabolites, including X11-deoxyprostaglandin F1α, X16-heptadecyne-1,2,4-triol, estriol, and prostaglandin A3. These metabolites are associated with lipid metabolism pathway according to the KEGG metabolic classification.^4^ The highest relative abundance of Quinella was observed in the HE group, indicating that an increase in dietary energy level can increase *Quinella* abundance, thereby potentially influencing serum lipid metabolism in yaks. *Selenomonas* was also found to be positively correlated with PC 36:5, X16-heptadecyne-1,2,4-triol, and prostaglandin A3. As *Selenomonas* ferment glucose to produce acetic acid and propionic acid (Zhao et al., 2022), and PC 36:5 serves as a choline storage molecule involved in the glycerophospholipid metabolism pathway (Athamena et al., 2011), these findings suggest that *Selenomona*s may contribute to the regulation of both lipid and glucose metabolism in rumen environment.

Additionally, *Succiniclasticum* in the rumen was positively correlated with *Prevotellaceae UCG-003* in feces. *Succiniclasticum* utilizes succinic acid to produce propionic acid (Liu X. et al., 2022), whereas *Prevotellaceae UCG-003* plays a role in the degradation of protein and starch. A negative correlation was observed between *Methanobrevibacte*r in the rumen and *UCG-005* in feces. Although both taxa are involved in fiber utilization, they produce different metabolic substrates (He et al., 2015; Zhang and Wang, 2018). The correlations between rumen and fecal microbiota indicate a functional and compositional linkage between these microbial communities, with distinct yet complementary roles in the digestive process of yaks. Notably, a significant relationship was identified between the relative abundances of *Prevotellaceae UCG-001* and *Clostridium sensu stricto-1*, underscoring the need for further investigation into their functional roles within the yak gastrointestinal ecosystem.

## 6 Conclusion

We combined data on weight, nutrient digestibility, rumen fermentation parameters, 16S rDNA analysis, serum biochemistry, hormone indicators, and metabolomics to investigate the impact of dietary energy levels on the house-feeding of yaks. In our study, the ME dietary energy level (NEg: 4.46 MJ/kg) can effectively improve the growth performance of yaks, enhance their energy metabolic level, promote the digestion of soluble carbohydrates, and ensure normal rumen fermentation. Meanwhile, the dietary energy level (NEg: 4.46 MJ/kg) significantly increased the relative abundance of *Proteobacteria* in the *maiwa* yak rumen. It also facilitated the growth of the fiber-degrading bacterium *UCG-005* in the feces, promoting gastrointestinal fermentation. Moreover, it improved the metabolism of lipids, amino acids, nucleotides, and vitamins in yaks, thereby enhancing their growth performance and ensuring their health. Our findings provide foundational data for the development of the yak industry in pastoral areas and offer new insights into the metabolites and microbial functions of yak.

## Data Availability

The original contributions presented in the study are publicly available. This data can be found here: https://www.ncbi.nlm.nih.gov/sra/PRJNA1298843.

## Footnotes

1
https://www.genome.jp/kegg/pathway.htm

2
https://hmdb.ca/metabolites

3
http://www.lipidmaps.org/

4
https://www.genome.jp/kegg/pathway.html
